# Experimental Investigations of Micro-Meso Damage Evolution for a Co/WC-Type Tool Material with Application of Digital Image Correlation and Machine Learning

**DOI:** 10.3390/ma14133562

**Published:** 2021-06-25

**Authors:** Yanling Schneider, Reiner Zielke, Chensheng Xu, Muhammad Tayyab, Ulrich Weber, Siegfried Schmauder, Wolfgang Tillmann

**Affiliations:** 1 Institute for Materials Testing, Materials Science and Strength of Materials (IMWF), University of Stuttgart, Pfaffenwaldring 32, D-70569 Stuttgart, Germany; Chensheng.xu@imwf.uni-stuttgart.de (C.X.); Siegfried.Schmauder@imwf.uni-stuttgart.de (S.S.); 2 RIF Institute for Research and Transfer e.V., Joseph-von-Fraunhofer Str. 20, D-44227 Dortmund, Germany; Reiner.Zielke@tu-dortmund.de (R.Z.); muhammad.tayyab@tu-dortmund.de (M.T.); Wolfgang.Tillmann@tu-dortmund.de (W.T.); 3 Material Testing Institute (MPA), University of Stuttgart, Pfaffenwaldring 32, D-70569 Stuttgart, Germany; Ulrich.weber@mpa.uni-stuttgart.de

**Keywords:** micro-computed tomography (μCT), digital image correlation, local strain map, damage evolution, machine learning

## Abstract

Commercial Co/WC/diamond composites are hard metals and very useful as a kind of tool material, for which both ductile and quasi-brittle behaviors are possible. This work experimentally investigates their damage evolution dependence on microstructural features. The current study investigates a different type of Co/WC-type tool material which contains 90 vol.% Co instead of the usual <50 vol.%. The studied composites showed quasi-brittle behavior. An in-house-designed testing machine realizes the in-situ micro-computed tomography (μCT) under loading. This advanced equipment can record local damage in 3D during the loading. The digital image correlation technique delivers local displacement/strain maps in 2D and 3D based on tomographic images. As shown by nanoindentation tests, matrix regions near diamond particles do not possess higher hardness values than other regions. Since local positions with high stress are often coincident with those with high strain, diamonds, which aim to achieve composites with high hardnesses, contribute to the strength less than the WC phase. Samples that illustrated quasi-brittle behavior possess about 100–130 MPa higher tensile strengths than those with ductile behavior. Voids and their connections (forming mini/small cracks) dominant the detected damages, which means void initiation, growth, and coalescence should be the damage mechanisms. The void appears in the form of debonding. Still, it is uncovered that debonding between Co-diamonds plays a major role in provoking fatal fractures for composites with quasi-brittle behavior. An optimized microstructure should avoid diamond clusters and their local volume concentrations. To improve the time efficiency and the object-identification accuracy in μCT image segmentation, machine learning (ML), U-Net in the convolutional neural network (deep learning), is applied. This method takes only about 40 min to segment more than 700 images, i.e., a great improvement of the time efficiency compared to the manual work and the accuracy maintained. The results mentioned above demonstrate knowledge about the strengthening and damage mechanisms for Co/WC/diamond composites with >50 vol.% Co. The material properties for such tool materials (>50 vol.% Co) is rarely published until now. Efforts made in the ML part contribute to the realization of autonomous processing procedures in big-data-driven science applied in materials science.

## 1. Introduction

In materials science and until now, four paradigms can be summarized for the guiding ideology [1]: the empirical trial-and-error method, the physical and chemical law, the computer simulation, and the big-data-driven science. Any later appeared paradigm can unify the previous ones. The above mentioned fourth paradigm, big-data-driven science, can combine the other three in the aspect of theories, experiments, and simulations. The source of data for materials science can be roughly categorized into four types: material properties from experiments and simulations, chemical reaction data, image data, and literature [2]. The total amount of collected data, e.g., generated by experiments and simulations, greatly surpasses the analyzing capability using traditional methods, e.g., manual handling of computed tomography (CT) images. For handling the source data, an automatic data sortation is a solution, e.g., using algorithms. As a further step and similar to human intelligence (i.e., artificial intelligence), such “algorithms” can, e.g., (i) learn from previous experience like human being and from the difference between source data and ground truth; (ii) develop more sophisticated procedures with higher time efficiency and more substantial stability; and (iii) discover or deduce new useful information, e.g., new materials as well as optimized parameters for simulations or image segmentations [3]. As a branch of artificial intelligence, machine learning (ML) has gained momentum in materials science. Learning methods can be divided into supervised learning, unsupervised learning, semi-supervised learning, and reinforcement learning. Deep learning in materials science includes convolutional neural network (CNN), recurrent neural network, deep belief network, and deep coding network. For model validation, many ML methods divide the original data into a training set and a test set and use the training set for model training and the test set for model validation. K-fold cross-validation [4] and leave-one-out cross-validation are common validation methods. Other existing methods are, e.g., repeating learning test cross-validation and bootstrap cross-validation. ML can effectively and efficiently identify patterns in large high-dimensional data sets, extract desired information and discover new laws. It is widely used in analyzing material property (degradation detection, nanomaterials analysis, and molecular property prediction) and discovering new materials (structure-oriented design, element-oriented design, inverse design, drug design, and quantum chemistry) [2]. Concerning the current work, the learning method belongs to supervised learning and further configurated by reinforcement learning [5,6]. In this work, U-Net [7] is used, which belongs to CNN in deep learning. U-Net possesses the function of reinforcement learning, before which supervised learning is the major process. Furthermore, it has the ability of argumentation of the masks to enrich the data base, including rotating, cropping as well as mirroring. The architecture of U-Net stems from fully convolutional networks (FCNs) [8]. The application of ML to materials science is a field often described as “materials informatics” [9,10]. Some review works about ML and materials informatics can be found in, e.g., [2,9,11].

For the development of the scientific field, already existing branches are actually as important as the new “comings”, all of which contribute to further progress of this field and mutually benefit each other. Simply, further research of existing branches ceaselessly adopts new tools or methods accompanying continuous advancements of technology and knowledge. The development of experimental techniques still plays a decisive role in materials science, e.g., providing the ground truth of ML method and delivering the “reality” for simulation results. To investigate material deformation behavior, micromechanical characteristics are vital to understanding the overall material performance. To achieve such knowledge, it relies upon experimental techniques of non-destructive and destructive nature. Such measurements can be, e.g., transmission electron microscopy (TEM), X-ray diffraction (XRD) and electron backscatter diffraction (EBSD) [12], as well as CT. Recently, 3D EBSD, e.g., in [13,14,15], and micro CT (μCT) combined with digital image correlation (DIC) have already been applied in investigations of material deformation behavior. For a relatively comprehensive study of material deformation behavior, studies based on multi-scales are preferred, since coupled effects of mechanisms on different scales determine the material deformation behavior.

In the field of material deformation recording, DIC is a non-contact optical measurement technique for full-field deformation measurement in experimental mechanics. This method employs image registration and tracking techniques for 2D and 3D measurements of changes in images, especially in micro and nano-scale mechanical testing for velocimetry, displacement, and strain estimation. The current work concentrates on the local displacements and strain maps. In DIC, images of an object in reference and deformed state are compared, i.e., correlated, for pixel movement in the selected region of interest to calculate the displacements [16]. The whole concept of DIC is based on two major assumptions [17]: (i) points on the image are directly related to corresponding points on the object in terms of motion development; (ii) to define the local image motion, accurate matching is performed based on adequate contrast of each subregion in the image. Small subsets containing information about the intensity values (i.e., speckle pattern) are selected from the reference image. In order to track the subset in the deformed image, a correlation criterion is defined. Moreover, a subset shape function is necessary to accommodate the subset’s changes in shape and orientation due to deformation. Pan et al. [18] listed some common correlation criteria and used first-order shape functions to depict the reference subset’s shape-change. Lu et al. [19] also proposed second-order shape functions in the context of DIC to address the complicated deformation behavior of the reference subset. Furthermore, during the deformation, the subset can also move to subpixel locations. The interpolation of the intensity values is needed for subpixel locations to achieve subpixel accuracy. To achieve this , the discrete data from the images is converted into continuous data with different interpolation schemes, e.g., bilinear and cubic spline interpolations [17]. The accuracy and validity of the DIC results are highly dependent on the speckle pattern in the image. Sutton et al. [17] listed some different methods of pattern application. The good selection of the subset size also depends on the speckle pattern’s quality, as subset size large enough to accommodate intensity variations is a pre-requisite for reliable results. However, a large subset size results in significant errors for displacement calculations [18]. In order to evaluate the quality, different parameters such as mean speckle size [20], subset entropy [21], and the sum of the square of subset intensity gradients [22] have been used in the literature.

Undoubtedly, material property investigation would also utilize the methods and techniques as mentioned earlier, such as simulation, ML, in-situ tomography, and DIC. As an important part of the material family, tool materials such as tungsten carbides (WC or WC-Co), Ti6Al4V, polycrystalline diamonds, boron nitrides, and high-speed steels exhibit properties with exceptional combinations of hardness, toughness, strength, and wear resistance. They are important for industrial manufacture and daily life. Hard metal inserts share half of the market for cutting tools [23]. For applications of hard metal components, such as cutting tools and drawing dies, the cemented WC-Co share about 98% of the market today [24]. For such materials, investigations on strengths, hardnesses and micromechanical properties can be found, e.g., in [25,26,27,28,29,30,31,32,33,34,35,36,37]. To further improve the hardness, diamonds can be added, and coatings, e.g., Ti or Ti-N coatings, can be used to enhance the bonding between diamonds and the base material (WC-Co). The diamond coating is also named as diamond film [32,33,34]. Some studies try to figure out the relation between the measured data and the predictions from simulations or existing theories, e.g., the Hall-Petch relation between grain size and strength [25,28]. The wear resistance and material behavior at high temperatures are also worth being researched, e.g., in [38,39,40,41,42]. The influence of phase transformation on wear behavior is another research topic [39]. Fatigue damage evolution is an important investigation aspect for tool materials, e.g., [43,44]. Some studies concentrate on microscopic issues, such as grain boundary energies, crystal orientations, and grain coatings [45,46,47]. A summary of the solubility of the WC-Co material system can be found in [48].

Using experimental tools available, the current work is aimed to investigate the damage mechanisms of commercial Co/WC/diamond metal matrix composites (MMCs). With 90 vol.% Co, the currently investigated composites, also a kind of WC-Co tool material, are much less studied than the same type of composites with more than 50 vol.% WC. A special characteristic of our MMC is that both ductile and quasi-brittle behavior are possible. Furthermore, commercial samples usually illustrate more physical, mechanical as well as morphological uncertainties. The question is, what would happen, when samples with quasi-brittle behavior possess simultaneously extreme morphologies, e.g., local particle cluster concentration and extraordinary large particle. To improve the material performance, it is significant to achieve knowledge about: (i) what are the dominant damage mechanisms of composites that show ductile and quasi-brittle behavior, respectively; (ii) does diamond particles or WC phase contribute more to the composite strength; and (iii) what is the difference of local strength/deformation in the matrix regions around diamond particles and large WC ones. Since damage evolution is mainly a result of coupled mechanisms on different scales, measurements are performed on submicron, micro, and macro scales to achieve relatively comprehensive knowledge. To answer the above mentioned (i)–(iii) questions, various experiments are performed, such as SEM, the nanoindentation, and the in-situ X-ray μCT combined with the DIC technique. Such testing methods/techniques make it possible to achieve material characteristics such as the local hardness distribution, the 3D real microstructure, and the 2D/3D strain maps. For handling the tomograms from μCT, the amount of work to sort the pixels in the tomographic images exceeds the limit of manual work. To greatly improve the time efficiency, ML is applied for image segmentation. To obtain local strain maps, the DIC method is applied. Concerning the WC-Co type tool materials, results for composites with WC >50 vol.% are coverd in most of the published data. Comparatively, significantly fewer works reported material properties for composites with WC <50 vol.%. Crostack et al. [49,50] measured the strain field and the crack development near diamond’s sharp corners on the meso scale for Co/WC/diamond composites (WC <50 vol.%) using μCT. The global strain reached about 10–11% under tension, i.e., ductile behavior. The composites in the current work showed quasi-brittle behavior, with the global strain reaching about 1.5–2.0%. The void growth and coalescence (forming mini/small cracks) dominate the damage development, i.e., not the crack development near the diamond’s sharp corner. ML applied for pixel sortation of tomographic images contributes to the realization of autonomous processing procedures in big-data-driven science applied in materials science. This work provides hints for the strengthening and damage mechanisms for Co-based WC-Co-type tool materials, and provides data and comparison possibilities for other similar studies.

It is noted that all the mentioned FE results about the studied Co/WC/diamond composites would be reported in another work in the near future.

## 2. Materials and Experiments

**Figure 1:**
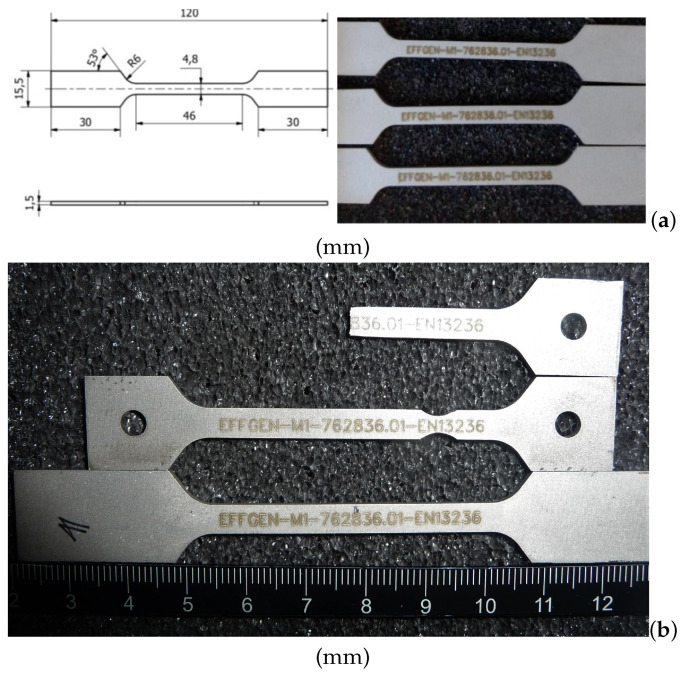
(**a**) Tensile sample geometry and purchased workpieces (showed quasi-brittle behavior) from company Effgen used in the current work; (**b**) samples including one after failure with another geometry together with the one in (**a**) for the same composite.

In the current work, two types of commercial Co/WC/diamond MMCs manufactured by the powder metallurgical process are investigated to achieve knowledge about their damage evolution. The Co phase with 90 vol.% can be taken as the matrix. In the application, the WC-Co together is often taken as the matrix and is commonly known as cemented carbides or sintered carbides with Co as the binder (WC more than 50 vol.%). The currently studied MMCs with 5 vol.% WC, similar to the ones with more than 50 vol.% WC phase, is also a kind of hard material. A producer will be suitable for material selection, who can produce test specimens according to individual specifications, and can also manufacture them in the required geometries. Company Effgen/Germany, with its F&E department, meets the above requirements and can also supply sample materials (Type-II in Table 1) with the same properties for further investigations. Figure 1a (left) shows the dimensions of the tensile specimens and manufactured workpieces from Effgen Figure 1a (right). This (Figure 1a presents the geometry of the mainly used samples. Figure 1b illustrates two samples with another geometry, including one after failure, together with the one shown in Figure 1a. Table 1 lists the composition and the particle mean size of the above mentioned two types of samples, which the manufacturer provides. Type-I in Table 1 behaved ductile, more information about which can be found in [49,50]. Type-II in Table 1 showed quasi-brittle behavior. The study about the type-II composite is the emphasis of the current work. As a comparison, selected deformation characteristics of type-I will be also mentioned here. Table 2 shows material properties, Young’s modulus, Poisson’s ratio, and thermal expansion coefficient, for the pure Co, the pure WC, and the diamond [49,50]. In Kim et al. [25], the measured Young’s moduli and the Poisson’s ratios are 714 GPa, 0.19 for WC and 211 GPa and 0.31 for Co, accordingly.

### 2.1. In-Situ μCT Equipment with an In-House-Designed Test Rig

**Figure 2:**
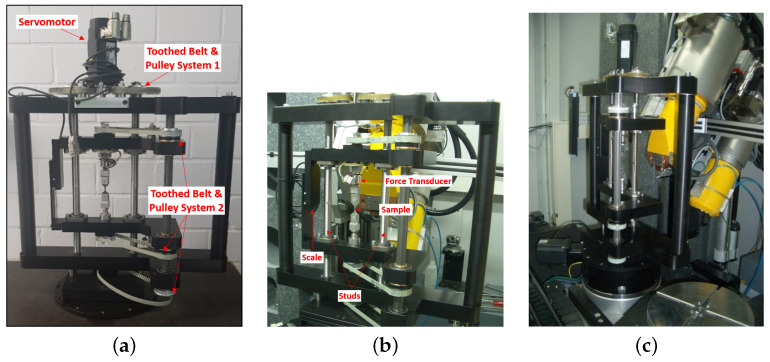
An in-house-designed test rig for in-situ X-ray microscopy (RIF institute, Dortmund, Germany): (**a**) test rig for in-situ X-ray CT; (**b**) inner platform of the test rig; (**c**) test rig installed in X-ray CT machine at the laboratory.

X-ray CT has the unique ability to observe the inside of the objects in 3D in a non-destructive way. This technique could exceptionally be helpful to observe the 3D damage evolution in the material under the condition that the X-ray CT scans are carried out during different loads. An in-house-designed test rig (RIF institute, Dortmund, Germany), which allows the rotation of the tensile specimen under load, was constructed for in-situ X-ray microscopy during tensile testing to achieve damage and displacement fields. Each component in this rig was individually designed by RIF and manufactured by different companies, and was completely assembled at RIF. The test rig consists of a synchronous servo motor coupled with two large studs through a toothed belt and pulley system (Figure 2a). The inner platform, consisting of the sample holder, force transducer, and a high precision absolute scale, rests on two large studs (Figure 2b). The large studs have left-hand threads for one half of the length and right-hand threads for the other half, ensuring that both ends of the inner platform move in the opposite direction with the rotation of studs during loading and unloading of the sample. The sample holding test rig of the inner platform is coupled to the rotating bed of the X-ray machine through another toothed belt and pulley system to rotate the sample during the X-ray CT scan (Figure 2c). The input and output of the whole test rig is controlled through the DOLi Test and Motion software at the personal computer connected to the motor and the sensors through serial communication ports and an electronic control box. The test rig was mounted in the GE X-ray CT machine available at LWT lab. The sample itself is mechanically loaded, utilizing the motor to drive the two spindles. The applied force is measured and recorded with the load cell. The elongation is also recorded through a displacement transducer. The sample holder can be rotated on the upper and lower holder and rotated through a laterally mounted axle and belt drive. In order to avoid the additional electric drive and angle sensors, the turntable GE vtome 240 computed tomography system was used for the drive. Thereupon, no change or adaptation of the CT measurement software is necessary. For the analysis of the samples, the XRadia 520 X-ray microscope from Zeiss [51,52,53,54], which allows a resolution of less than 1 μm, was used in addition to the μCT system. One sample was firstly loaded in tension with a conventional traction machine until it broke. Thereafter, using the obtained tensile stress-strain curve, load points for the CT images (for another sample or measurement) were then selected from ranges of elastic and plastic loadings.

### 2.2. Monotonic Tensile Loading and In-Situ Microtomography

**Figure 3:**
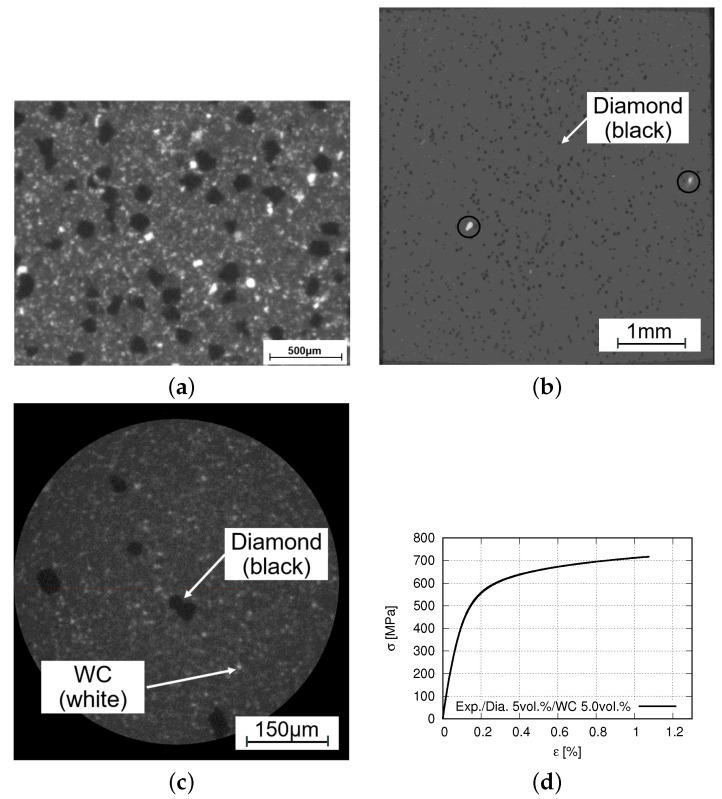
Real 2D microstructure cut-outs and stress-strain curve of Co/WC/diamond composites: (**a**) an SEM image [49] (type-I, ductile behavior); (**b**) one tomographic image with a pixel resolution of 5.2508 μm/pixel (scan-A, type-II, quasi-brittle behavior), where two big WC particles are marked in circles (mean size 3–5 μm); (**c**) one tomographic image with a pixel resolution of 0.6715 μm/pixel (scan-B, type-II, quasi-brittle behavior); (**d**) a global tensile stress-strain curve (type-II, quasi-brittle behavior).

Figure 3a, an SEM image, presents the type-I composite’s microstructure, which depicts a ductile material behavior with a global fracture strain of about 10% [49]. Figure 3b,c illustrate two microstructure cut-outs of the type-II composite in X-ray images, where the pixel resolutions are 5.2508 μm/pixel and 0.6715 μm/pixel, respectively. In the following, the tomography scan with a pixel resolution of 5.2508 μm/pixel is called “scan-A” (Figure 3b), and the other one mentioned above is called “scan-B” (Figure 3c). The detected size spectrum of WC particle diameters from measurements covers about 1 μm to about 150 μm. In Figure 3b, two big WC particles are marked with two circles. Comparatively, the WC particles with relatively small sizes can be found in Figure 3c.

The type-I composite’s fracture stress, also the ultimate tensile stress, reached approximately 630 MPa, and the fracture (true) strain about 10% [49]. Concerning the type-II composite, all four tensile tests gave macroscopically quasi-brittle behavior. The fracture (true) strain is about 1.2–2%, and the ultimate tensile stress is about 720 MPa. A global stress-strain curve of the type-II material is illustrated in Figure 3d.

**Figure 4:**
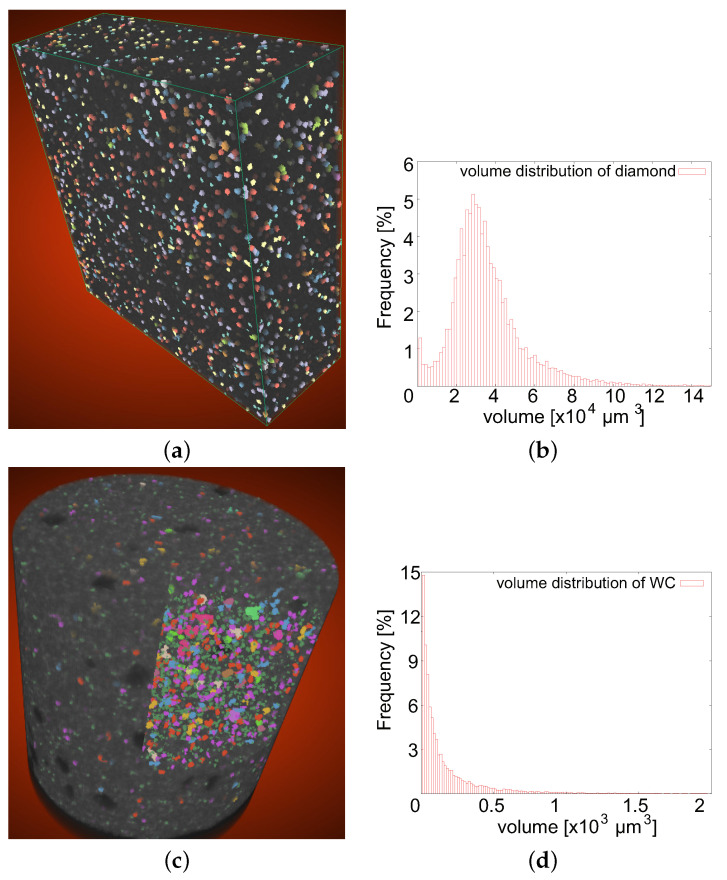
The particles and the size distribution of the diamond and the WC phase (type-II quasi-brittle behavior): (**a**) diamond particles shown in color and reconstructed from tomographic images (scan-A); (**b**) the corresponding diamond size distribution with a mean volume of about 3.6 $\times 10^{4}$ μm${}^{3}$; (**c**) WC particles shown in color and reconstructed from tomographic images (scan-B); (**d**) the WC size distribution corresponding to (**c**) and with a mean volume of about 2 $\times 10^{2}$ μm${}^{3}$.

The 3D real microstructure of the Co/WC/diamond composite was represented with the aid of the in-situ X-ray μCT. Such a tomographic scan is done perpendicular to the tensile loading direction, i.e., the loading direction is parallel to each tomographic image. The WC particles, which are small compared to diamonds, can be made visible by making the Co matrix phase thoroughly transparent. Figure 4a covers a volume of 3.5 × 3.7 × 1.3 mm${}^{3}$, which includes more than 13,400 diamond particles. After recalculation of the scanned tomographic images, the mean volume of diamond particles (scan-A) is 3.67 × 10${}^{4}$ μm${}^{3}$ (Figure 4a), which corresponds to a diameter of about 50 μm. Figure 4b shows the corresponding diamond volume distribution. In Figure 4c, the WC particles have a mean size of about 7 μm in diameter. The total scan volume presented in Figure 4c is about 0.159 mm${}^{3}$, which includes about 13,400 WC particles. Only WC particles with a volume higher than 9.99 μm${}^{3}$ are considered in Figure 4c. In this case, the possible noises are excluded in the tomographic images. It is reasonable that the mean size of WC particles in the measurement (Figure 4c) is higher than the value given by the manufacturer (Table 1). Figure 4d illustrates the volume distribution of the detected WC particle. The remaining WC particles, i.e., the undetected and small ones, have sub-micron sizes and are very important for the particle strengthening effect in MMCs, since, according to Hall-Petch effect, smaller sized particles usually contribute more to the material strength compared to bigger ones.

### 2.3. Damage Distribution

**Figure 5:**
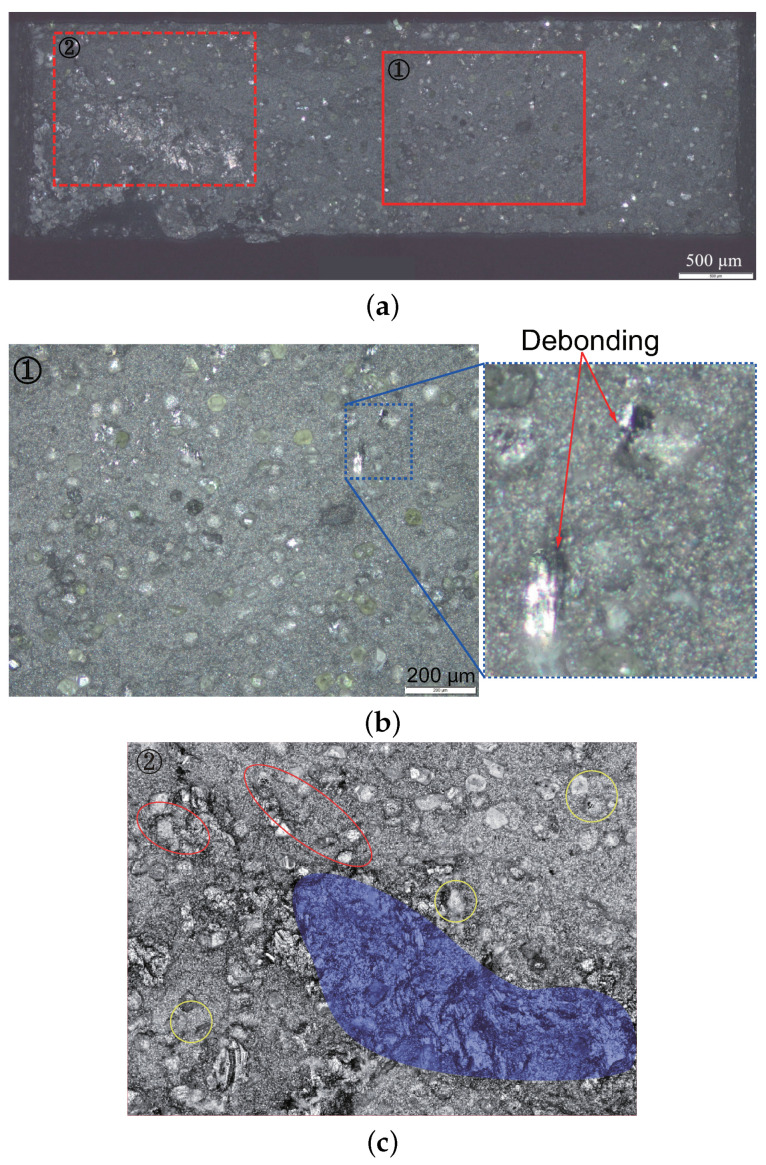
Fracture surface (type-II quasi-brittle behavior): (**a**) the whole fracture surface, where the rectangles in solid and dashed lines are shown in (**b**,**c**) at higher magnifications, respectively; (**b**) zoomed-in view with more recognizable diamonds; (**c**) zoomed-in view, where clusters of diamond particles amount to about 12 vol.% (5 vol.% in manufacture).

To find out the relation between damage development and microstructural features, such as the morphology and the local damage distribution, light microscope photography was used. Figure 5a demonstrates the entire fracture surface of the type-II sample, where the rectangle area marked with a solid line is shown in an enlarged view in Figure 5b. Figure 5c presents the rectangle area marked with a dashed line in Figure 5a, where artificially added colored circles/ovals partly mark the damages. The contrast has been configured for Figure 5c, aiming to illustrate the damages more visibly [55]. The diamond volume fraction amounts to approximately 12 vol.% in Figure 5c. The main debonding/crack causing the fatal failure should be formed in this region (Figure 5c) and the pattern of the debonding/crack distribution should be much local morphology dependent. The possible fatal damage could be particle debonding, particle fracture, matrix fracture, or main crack(s). Compared to regions shown in Figure 5b, much more damages, including particle debonding and mini cracks as well as crack connecting, are detected in the region presented in Figure 5c. In Figure 5c, the yellow circles denote the void/debonding between diamonds and the matrix, while the red ovals mark the void/debonding connection. No obvious debonding between Co and WC is detectable. Regions enclosed by the purple color present some complex damage distributions. The original fracture surface in the corresponding area (purple colored area) is partly very coarse. It is supposed that some parts of these regions show no damage evolution since such parts broke suddenly at the fracture time point. In other words, it is difficult to accurately analyze the damage evolution characteristics of the purple colored regions, as shown in Figure 5c. From the observed damages in all of performed experiments, particle fracture is observed for neither WC nor diamond. Also, no matrix fracture is detected in Figure 5a. When net-like cracks appear in the matrix, it is considered as matrix fracture. The connections (can be taken as cohesiveness) between WC and Co is very stable during loading. Comparatively, diamonds have a much poorer connection with Co. Still, diamond possesses a much larger mean size than WC. Debonding appears on larger-sized particles more easily than on smaller-sized particles. Based on these, the WC debonding is not considered here. Still, some void connections are observed, i.e., cracks are formed (Figure 5c). Limited by experimental data, it is not easy to fix the fatal failure due to diamond debonding or the formation of cracks. From the above discussion and since the sample showed quasi-brittle behavior, the dominant damage mechanism should be the diamond particle debonding and void/debonding connecting.

Due to limited literature about Co/WC/diamond composites with Co >50 vol.%, no similar knowledge about the primary damage mechanisms could be obtained from other works. Comparison with findings for composites with <50 vol.% Co is not suitable since the currently studied composites are different from those. Further investigation is necessary to achieve a more fixed and detailed understanding of the fatal failure mechanism in WC/Co composite with Co >50 vol.%.

### 2.4. Nano Hardness

The yield stress reaches around 450 MPa from the global stress-strain flow behavior for the type-II composite. Pure Co has a yield limit of about 220 MPa. From 220 MPa to about 450 MPa, such a high strengthening effect should result from hard WC particles, diamond, and lattice structure. During manufacturing, there exists a certain amount of solubility, which means that WC is partly dissolved in the Co lattice, e.g., forming a new type of Co-WC lattice [48]. Usually, locations with higher hardness values also possess higher strength values, e.g., higher Young’s moduli. We are interested in the local matrix hardness/strength alteration as a function of the distance to diamonds. If the yield limits around some WC particles are higher than those around some diamond particles, it provides evidence that the WC phase possesses a higher mean stress than the diamond phase.

**Figure 6:**
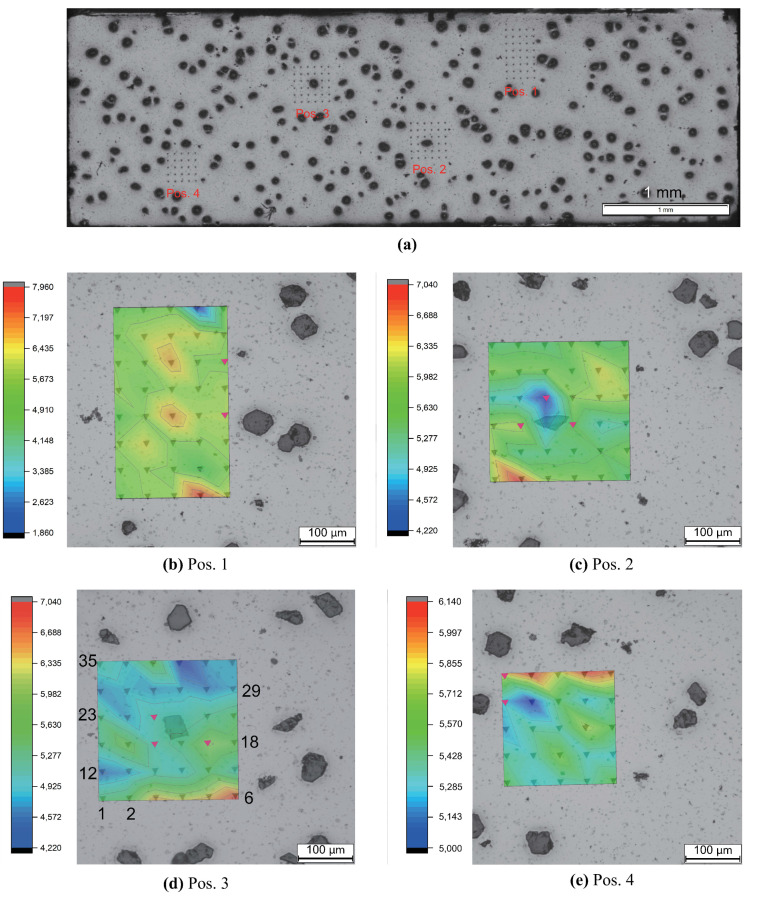
Hardness distribution from HV0.3 nanoindentation tests after graphitization of diamonds (type-II quasi-brittle behavior): (**a**) the four measured positions (Pos.1 to Pos.4) on the surface; (**b**–**e**) the hardness distributions correspond to Pos.1–Pos.4.

Since diamond particles in the samples may damage the diamond tip of the indentor, the graphitization of diamond particles is necessary to protect the indentor. The graphitization is done at a temperature of about 800 ${}^{\circ }$C after the tensile loading. Figure 6 illustrates test point positions in microstructure cut-outs and local hardness distributions. For each measured position, the numbering of the testing points starts from left to right in the bottom line, consecutively from right to left in the second bottom line. No measured point coincides with any diamond position. Figure 6d presents an example of such numbering, and there are 35 points in total, i.e., not 36 points. All the 134 measured points (Figure 6b–e) possess a mean hardness with a value of 5.72 GPa (drilling depth in a range of [500–1500] μm) and the mean Young’s modulus of about 200.42 GPa (range [103, 235.6] GPa). The graphitization causes hardness reduction. It is assumed (assumption-A) that this hardness reduction is homogeneous for the whole sample on the microlevel. It implied that no local regions showed a higher hardness reduction than any other regions due to this graphitization. If a local region shows a higher mean hardness value than any other after the graphitization, this characteristic also exists before the graphitization. From Figure 6a–e, the following properties can be identified: (i) the inhomogeneity of the local hardness values that vary between 1.83–7.95 GPa; (ii) the variation of the hardness value is about 334% ($\frac{\left|1.83-7.95\right|}{1.83}\approx 334\%$); (iii) matrix regions near a diamond particle with relatively lower hardness values; (iv) the highest hardness value located in regions between diamond particles (away from diamond particles); this is presented by all the four indentation positions shown in Figure 6b–e. Concerning the item (iii) mentioned above, diamond particles do not contribute much to enhancing the hardness of matrix regions nearby. Comparatively and as given in item (iv), higher hardness values are shown in some regions in the matrix (away from diamond particles), locations of which depend on the local morphology and the WC particle distribution.

**Figure 7:**
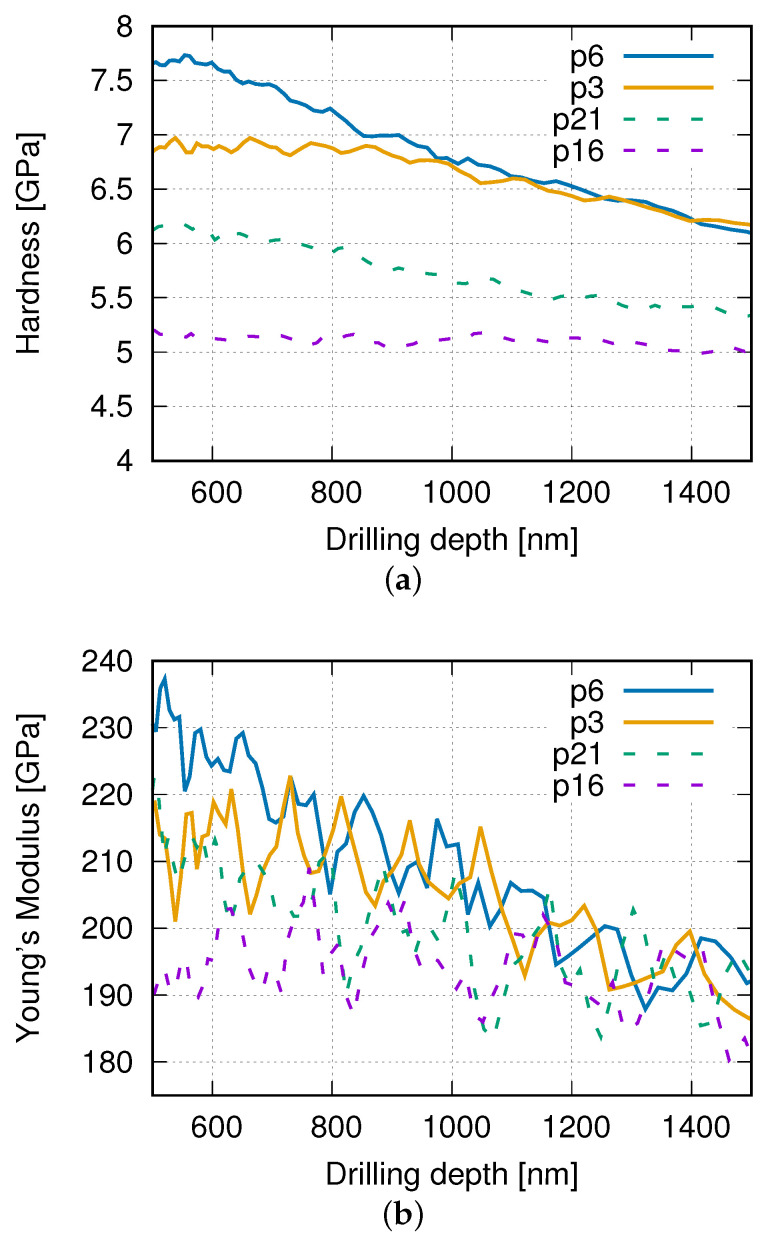
Measured values according to the drilling depth from HV0.3 nanoindentation tests after graphitization of diamonds (type-II quasi-brittle behavior, point number shown in Figure 6d): (**a**) hardness; (**b**) Young’s moduli.

Figure 6 presents the hardness distribution on the measured surface in the (Co-WC) matrix. To obtain knowledge about the local properties in the third direction, Figure 7a,b illustrate the measured curves of the local Young’s modulus and the hardnesses according to the drilling depth for some selected points in Figure 6d, respectively. The whole drilling depth is 2000 μm. The range of [500,1500] μm is presented here since the facility is not stable enough to given accurate measured values outside this range. Point 16 and 21 in Figure 7 locate very close to the diamond, and point 3 and 6 possessing the high hardness values are far away from the diamond (for point positions refer to Figure 6d). Through the whole depth, local matrix points with large distances to the diamond show apparently higher hardness values than points close to diamonds (Figure 7a). The measured 134 points (Figure 6b–e) are categorized into two groups, points far away from (G-F) and those closed to (G-N) diamonds. If the distance between the measured point and the nearest diamond border is less than that between two measured points (about 80 μm), this measured point belongs to the group G-N, which contains 10 points. Figure 6b–e marked these 10 points in red. The mean value of the hardness (average hardness value for the drilling depth in 500–2000 μm) in G-N is about 5.659 GPa. Comparatively, this value in G-F is 5.725 GPa. The Young’s-modulus curves (Figure 7b) demonstrate a similar tendency in general, even though fluctuations exist. Here, fluctuation means the other way around trend , i.e., the increment of Young’s modulus according to the increment of the drilling depth in some subregions. From the comparison of Figure 7a,b, the inhomogeneity evolution of the local elastic property illustrates much larger fluctuations than the hardness in the Co-WC matrix. i.e., the local elasticity field is highly heterogenous. According to the drilling depth, the local properties also prove that WC phase possessing higher mean stress than diamonds is quite possibly correct.

The same nanoindentation test will be executed on an original sample without graphitization to test the assumption mentioned above experimentally. It means, more or less, there is a danger that the indenter would be damaged if the diamond tip of the indenter meets the diamond in the composite. More troubles lie in the diamonds in the composite under the surface than the ones on the surface. For a successful test, results will be shown in a consecutive report, i.e., in the FE simulation part. If a relation of the measured local values were established between original samples and graphitized ones, it would be beneficial to further study. A μCT test before the nanoindentation would minimize the danger mentioned above. On the other hand, the selected measured positions would be more purposeful and helpful to deduce more concrete conclusions.

### 2.5. Strain Maps Measured by Digital Image Correlation Technique

**Figure 8:**
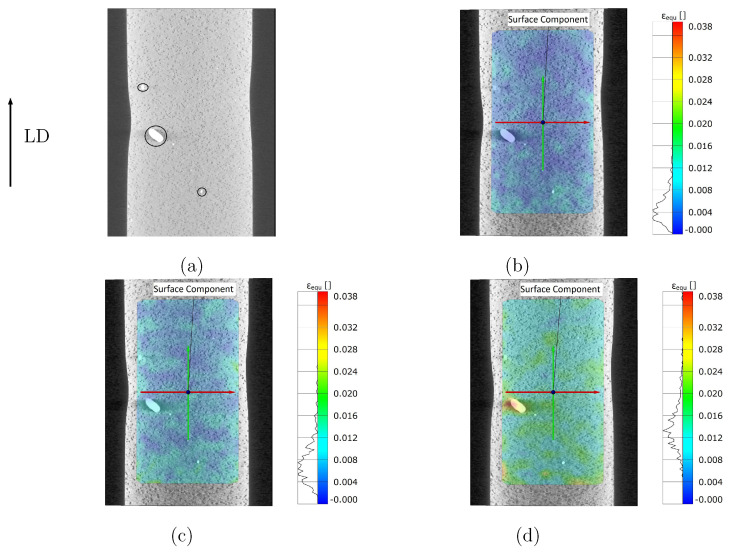
Stress controlled tensile loading: (**a**) in-situ X-ray μCT images at the load of 0 N; (**b**–**d**) strain maps (von Mises) calculated by digital image correlations (GOM [59]) at loading 2000 N, 3800 N and 4600 N, respectively.

Considering the present case, the Co/WC/diamond sample’s damage development was recorded using in-situ X-ray μCT during the tensile test. A force-controlled tensile test was carried out in three steps with forces of 2000 N, 3800 N, and 4600 N along the Y-axis (arrow in Figure 8 presenting the loading Y direction). The applied force reached about 5000 N at the fatal failure of the sample. A μCT-scan was carried out at the beginning (0 N) and at each step before further loading on the sample. A slight area reduction at the center of the sample was artificially introduced before the test to guarantee that the fracture surface remained within the scanning focus. As the sample moved during the imaging process, the misalignment among different scans existed for the extracted image slices. A proper geometric alignment of the image slices was required to ensure the accuracy of subsequent DIC analyses. The image slices at different loads were firstly aligned to the reference image (0 N) using Dragonfly software [56] through manual translation and rotation tools. To further improve the alignment, the feature-based automatic alignment option by Dragonfly [56] was applied. In order to derive the local strain maps, the μCT-scan image slices at different loads were analyzed by the DIC technique using five different software/codes. The strain maps calculated by all the five software/codes were almost similar, a part of which will be presented in the current work. If there is no other explicit notation, the measured local strain field is presented as the von Mises equivalent plastic strain $\varepsilon_{equ}$.

The current work mainly presents the 2D DIC analysis of the acquired image slices, where 3D ones will be reported in a further work. 2D DIC analyses are carried out by applying five different software, namely DICe [57], Ncorr (MATLAB) [58], GOM Correlate [59], VEDDAC [60], and an in-house code developed by us, RIF institute, Dortmund, Germany. Open access is available for DICe and Ncorr/MATLAB, while GOM and VEDDAC are commercial software.

**Figure 9:**
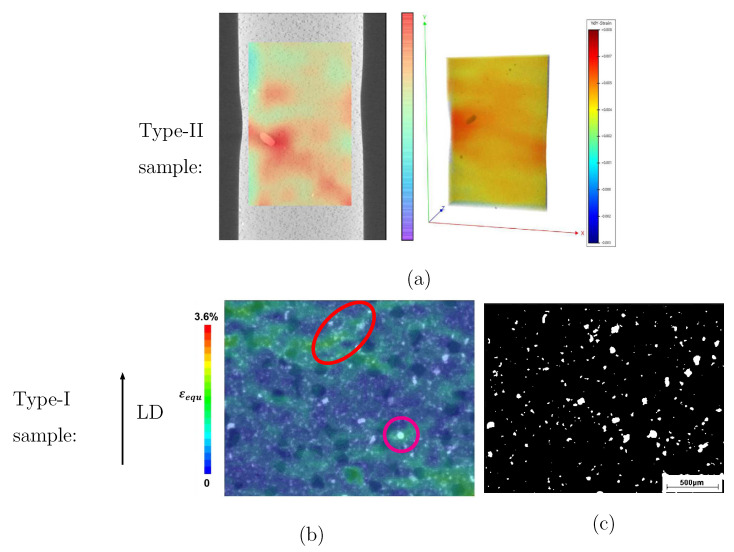
Measured local strain distribution: (**a**) results of image correlations from RIF e.V. (left, 2D DIC) and from VEDDAC [60] (right, 3D DIC), which is proved by Chemnitzer Werkstoffmechanik GmbH; (**b**) the measured local equivalent plastic strain $\varepsilon_{equ}$ [49,50] for the real microstructure shown in Figure 3a, where the arrow shows the loading direction; (**c**) the segmented WC phase of (**b**).

Figure 8a illustrates the initial stage of the measured mesostructure (surface of the sample) at 0 N. In Figure 8a, two abnormally large WC particles (mean size: 3–5 μm) are marked by two small circles. A few abnormally large-sized WC particles existed in the studied samples. The larger circle in Figure 8a enfolds an extraordinarily large WC particle (WC-A, its recalculated diameter being about 620 μm and its area ≈0.3 mm${}^{2}$). Even for commercial samples, a WC particle as large as WC-A does not normally appear. No such vast WC particle as WC-A appeared in other measurements, which means that the WC-A’s existence is by chance. Figure 8b–d, representing the 2D DIC results calculated by GOM Correlate, demonstrate the strain maps at 2000 N, 3800 N, and 4600 N, respectively. The heterogeneous local strain distribution is evident in the strain maps. The strain map pattern is local morphology dependent, especially in the case of large WC particles. Around diamond particles, no apparent high strain values exist. As mentioned in Section 2.4, high strain and stress values usually coexist in the same positions in the material. Based on FE simulation results, this coexistence is also true for the current studied Co/WC/diamond tool material. Another result of FE simulation illustrates that the WC phase possesses a higher mean stress than the diamond, to prove which a direct local stress measurement is not available now. The strain map in Figure 8 should be an experimental proof for the numerical result that WC phase contributes more to the strength of the composite than diamonds. Figure 9a presents the calculated strain distribution in the loading direction by self-developed code (left) and by VEDDAC (right), respectively. Since the strain values calculated by both codes are approximately the same (Figure 9a), it means that the self-developed code is applicable for DIC calculation. Figure 9a (left) is from a 2D DIC analysis, and Figure 9a (right) is from a 3D DIC analysis. In comparison to the strain maps of type-II composites with quasi-brittle behavior, Figure 9b [49,50] illustrates the measured local strain distribution of type-I composite with ductile behavior superimposed on the real microstructure as shown in Figure 3a [49,50]. For a clear visualization, Figure 9c shows the segmented WC phase corresponding to the microstructure in Figure 9b. From the strain map in Figure 9b, the main characteristics of the measured $\varepsilon_{equ}$ are: (i) appearance of the shear bands oriented in about 45${}^{\circ }$ to the loading direction, e.g., the region marked by the red oval; (ii) highly inhomogeneous local strain distribution, i.e., the dependence of strain map pattern on individual local morphology; (iii) generally, no detection of high strain values around diamond particles; (iv) around large WC particles or in regions with relatively high WC density, appearances of high strain values, e.g., regions marked by the magenta circle and by the red oval. Concerning point (iv), a possible reason is that such regions locate in the range (that is) under the influence of shear band. As high strain values are high deformation or high deformation gradient dependent, the high strain, and the high deformation gradient values are coincident. In such regions, since the material properties and the local morphologies change drastically, the compatibility and equilibrium conditions to be fulfilled are critical for the matrix, leading to a high deformation gradient. One of the further works will focus on calculating the strain maps using 3D DIC, which will provide more data to analyze the local deformation behavior.

## 3. Tomographic Image Segmentation

Besides recording damage and strain developments, the in-situ CT images also provide data useful for FE simulations, e.g., real 3D microstructures. Furthermore, displacements measured at different loading stages enable calculations with real boundary conditions (BCs), which actualizes more accurate numerical predictions than those with artificial BCs. At the moment, the 3D real microstructure is needed for an FE micromechanical damage prediction. In order to improve the time efficiency, it is advantageous to accomplish the image segmentation possibly automatically. In this section, Python codes, which mainly interpret the packages in the image processing and ML branch, are used, including the applied open-access ones.

### 3.1. Denoising Technique

**Figure 10:**
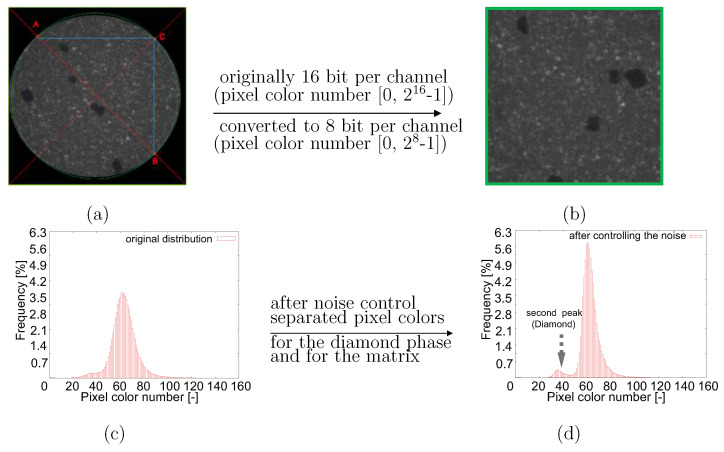
Tomographic image handling (scan-B, type-II quasi-brittle behavior): (**a**) extraction of a possible large square from each tomographic image with red-green-blue pixel color; (**b**) image with greyscale color; (**c**) noise exists in original images; (**d**) after noise control, pixel colors presenting diamonds separated from those presenting the matrix.

**Figure 11:**
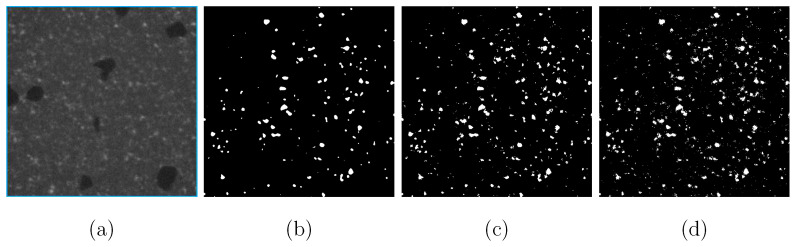
(**a**) An original CT image with noises after the extraction of the inscribed square out of a circle; (**b**–**d**): final denoising results of the WC phase using median, Gaussian, bilateral denoising methods [61], respectively.

Material structures are presented in a circle in each square-shaped source μCT image with black color as the background (Figure 10a, scan-B as mentioned in Figure 3c and Figure 4c). Three channels (red-green-blue/RGB) with 16 bits per channel describe the original tomographic images, i.e., the pixel number in the range of $\in [0,2^{16}-1]$ for each color (three numbers per pixel for the RGB format). A Python program cuts the maximum square area from each tomographic image (blue square in Figure 10a) and converts the three-channel RGB into one-channel grayscale with 8 bits per channel (Figure 10b), i.e., the pixel color number $\in \left[0,2^{8}-1\right]=\left[0,255\right]$ (one number per pixel for grayscale). To extract an inscribed square of the scanned circle area, Figure 10, the first image in the CT image stack (closest to the X-Ray source), illustrates the extraction process: (i) to draw the two red diagonal lines of the whole image; (ii) from the image vertex to the center along these two diagonal lines, to find three points/pixels, e.g., A, B, and C, which are the first non-black points; (iii) using the three points found from (ii), to determine the green circle enclosing the round scanned area; (iv) in the green circle, to extract its inscribed square shown in blue. After the fixation of the blue square in Figure 10a, it is easy to extract the corresponding squares from all other CT images. Noises impede further image processing (Figure 10b). Due to the thermal noise caused by the incident electrons, the image quality of the CT images is not good enough for direct image segmentation. Compared to lighter areas, the dark areas will be more severely affected. The salt-and-pepper noise is the major one in the current case and the image smoothing technique is applied for denoising. Such denoising belongs to part of the digital image analysis process for the presented tomograms. After the histogram analysis, only one peak, as shown in Figure 10c, could be recognized. The diamond pixel colors are completely hidden in the noise and mixed with the matrix. To separate the pixel color of the matrix from the ones of the diamonds, a Python program including open access algorithms [61] executes a noise-control process. Figure 10d denotes the histogram of the pixel-value distribution after denoising. After noise control, pixel colors presenting diamonds are separated from those presenting the matrix. Denoised images provide the possibility of ensuring the accuracy of the next step in image processing, i.e., using ML or the threshold method [62] for image segmentation. For the WC phase, the median filter [61] processed denoising. Figure 11a demonstrates an original CT image cut-out (same size as the blue square in Figure 10a). In order to segment the WC phase and to achieve trustable results, three different kinds of linear filters [61], median, Gaussian and bilateral, are used for the denoising, the results of which are presented in Figure 11b–d, respectively. The median filter and the Gaussian filter deliver approximately the same result and are taken as better ones compared to the bilateral filter. The better performance of the median and Gaussian filter is more obvious in Figure 14.

### 3.2. Machine Learning: U-Net

At its most basic, ML is the practice of using algorithms to parse data, learn from it, and then make a decision or prediction about the given purpose [63]. In ML, after the data collection, the next step is extracting suitable characteristics (features) from the raw data to enable its application, i.e., feature engineering. Shallow feature engineering, the traditional ML method, requires manual work, the amount of which can be huge. Shallow learning includes, e.g., support vector machine, decision tree, and artificial neural network, mainly linear classification. The more recently advanced deep learning has eliminated the necessity of manual work.

U-Net learns semantic segmentation in an end-to-end, pixel-to-pixel setting and needs very few annotated images (approximately 30 per application), i.e., another advantage of using U-Net. These 30 original images, together with their corresponding manually segmented images for diamonds (totally 30 × 2 images), are put in the label for the training of U-Net. I.e., two characteristics of U-Net to train FCNs end-to-end are [64]: (1) the first work for pixel-wise prediction; (2) from supervised pre-training. Using U-Net to segment images is very time-efficient and has a high accuracy. In the current case, the training time is about 1 h, where 30 images as the ground truth for the training are segmented manually. After training and for the segmentation of the diamond phase from 723 CT images, it takes about 40 min.

**Figure 12:**
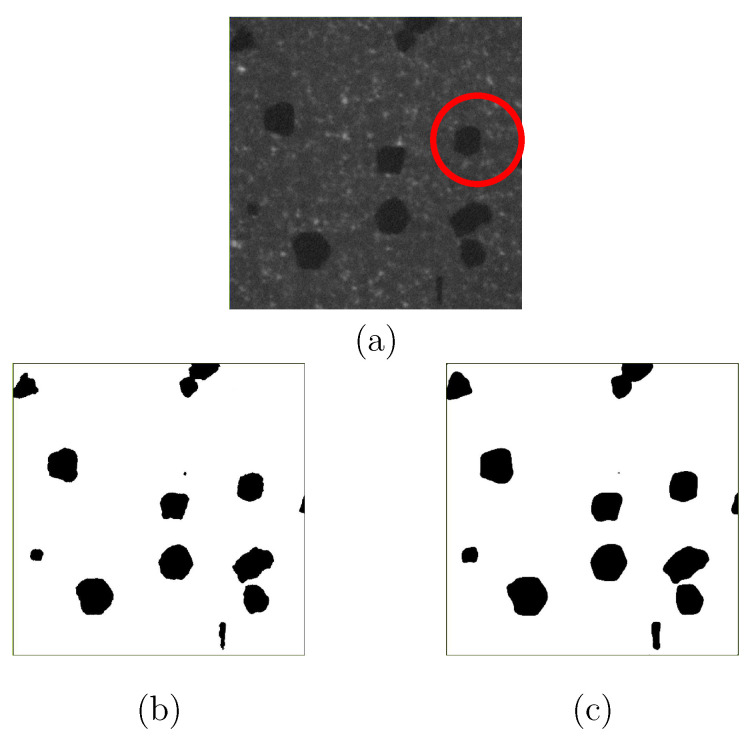
Image segmentation for diamonds: (**a**) an original CT image; (**b**) median filter with an aperture linear size of 7; (**c**) U-Net (CNN in ML).

**Figure 13:**
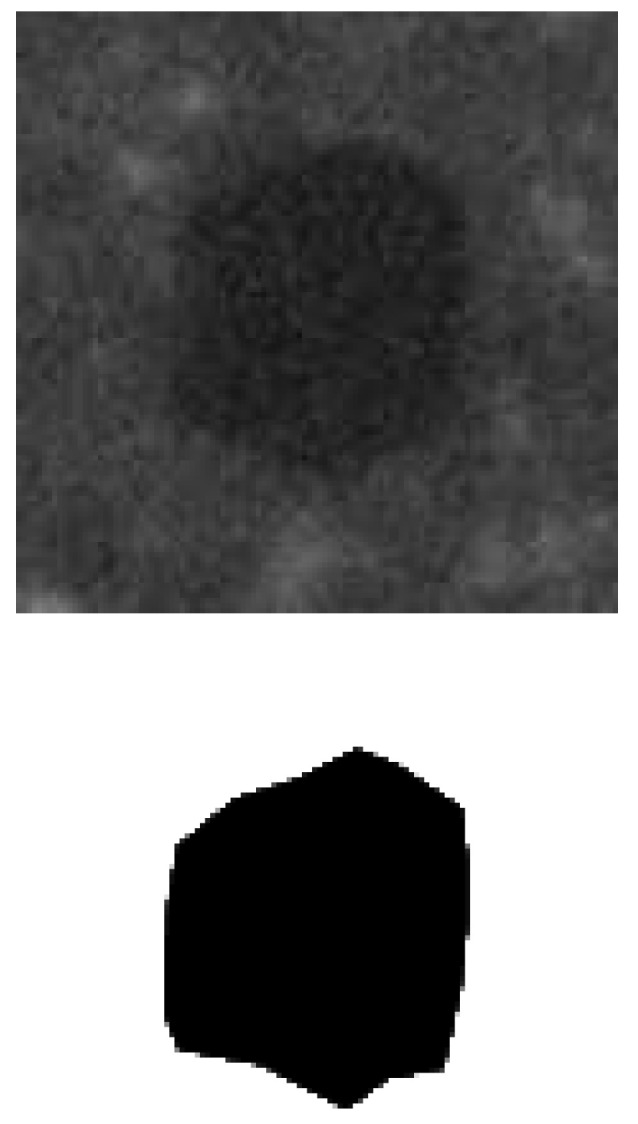
U-Net: an example of reinforcemen learning to capture the feature details of the diamond marked with a red circle in Figure 12a.

The subsequent work would provide the ground truth for the segmented results, e.g., using X-ray diffraction (XRD) technique or 3D EBSD, which will result in more accurate local features. I.e., due to limited time and facilities, no ground truth is available from experiments now, only the ones from manually segmented images. Here, it is assumed that the inaccuracy introduced by manual pixel sortation is negligible. To ensure a trustable segmentation result, the threshold method also segmented diamond particles for all the images. Both methods, ML and threshold, lead to approximately the same outputs. However, comparatively, U-Net (ML) can capture features in more detail. Figure 12a is a selected CT image, the diamond segmentation of which is compared in Figure 12b,c by using the median filter (threshold) method and the U-Net (ML) method, respectively. Figure 12b is the output with an aperture linear size of 7. Obviously, the U-Net process delivers better results since it captures the detailed features of the target object and can result in more smooth boundaries of objects. Diamond particles have polyhedron shapes, and each surface is smooth, i.e., not zig-zag curved. The reinforcement learning ability of U-Net is demonstrated in Figure 13 to capture the feature details. It is noted that the U-Net method in ML is not suitable for the segmentation of WC phase in the current images since WC particles are too small to achieve accurate training data. Meanwhile, the boundaries of WC particles are too ambiguous, which leads to high inaccuracy in ML. For the usage of U-Net, its advantage would be more pronounced when the images include more different objects compared to the current case with one object: diamond.

### 3.3. Result Comparison of Different Image Segmentation Methods

**Figure 14:**
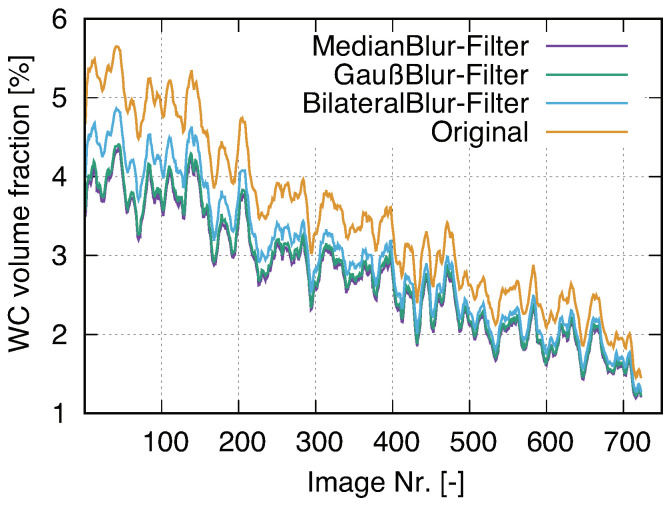
Comparison of WC phase area fractions in each μCT image among the original and filtered ones, where the filtration is done by three different methods [61].

To obtain some knowledge of the denoising or segmentation quality, three different filters, median blur, Gaussian and bilateral, are applied for WC phase segmentation in the CT image processing. The detected WC volume ratio is variable from one CT image (each pixel corresponding to one voxel) to another and used as the criterion for the quality evaluation of filters. For a good segmentation quality, the spectrum of the variance should be as low as possible since the WC particles are relatively very fine and continually distributed (i.e., the volume alteration from a local place to another should be small). Figure 14 illustrates the comparison results. The median filter and the Gaussian filter deliver approximately the same behavior for the WC volume variance and are taken as the better ones compared to the bilateral filter. It is pointed out that the original WC volume variance (without applying filters) is taken as possibly wrong (Figure 14), since the chance is nearly zero for WC phase that its volume fraction is locally higher than 5vol% (real composition). About half of all the WC particles are submicron-sized, i.e., the current CT scan cannot detect such WC particles. Moreover, WC particles are very fine, which leads to a reduced possibility of very high local concentration. These two reasons lead to the expectation that a local WC volume higher than 5vol% is nearly impossible. One common characteristic of the three curves is that the WC area fraction shows decreasing tendency from the first ( closest to X-ray source) to the last image (Figure 14), even though fluctuations are also presented in some subranges. This tendency is considered to be an artificial effect due to the divergence of the X-ray beam, which is more serious with the increasing scanning depth into the material. As a result, the diameter of the X-ray beam increases with the increasing scanning depth, which makes it more difficult to detect small WC particles. As a visible illustration of this effect, the detected WC particle area fraction decreases according to the increment of the scanning depth.

**Figure 15:**
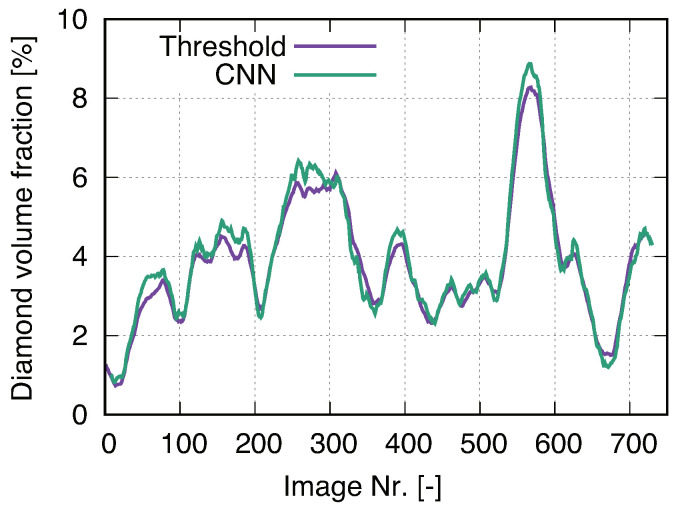
Comparison of diamond area fraction in each μCT image after the segmentation: threshold method [61] vs. U-Net/ML [7].

Two methods, threshold, and U-Net/CNN, are used to segment diamond particles. Figure 15 shows their segmentation qualities, where a relatively similar behavior of the volume variance is presented. The fluctuation of the volume ratio obtained by U-Net is slightly higher than the one obtained by the threshold method. It means U-Net can detect more detailed features of diamond particles, which are surrounded by a similar colored matrix. Furthermore, and from Figure 15, the diamond particles detected by U-Net are smoother ( closest to reality) than those detected by the threshold method. In conclusion, U-Net is the preferable method for the segmentation process of the diamond phase. Figure 15 provides information of the volume fraction of diamonds, which is very useful for selecting representative microstructure cut-outs for FE simulations.

## 4. Conclusions

The aim is to experimentally and numerically study the local damage evolution for commercial Co/WC/diamond MMCs, and the current study concentrates on the former. With 90 vol.% Co, such tool materials are not often reported in the literature. One particular characteristic of such composites is that their deformation behavior can be either ductile or quasi-brittle. The sample with quasi-brittle behavior possesses a mean size ratio of diamond: WC about 10:1, which is about 20:1 for the composite with ductile behavior. Cross-dimensional measurements are performed in 2D and 3D, e.g., macro stress-strain flow behavior, strain maps on mesoscale, and nanoindentation. The local strain field is measured by in-situ μCT combined with the digital image correlation technique, where five different codes/software are used to guarantee the accuracy of the calculated strain maps. Currently, strain maps are calculated based on 2D and 3D DIC. For an efficient and accurate data-processing of tomographic images (image segmentation), a machine learning method, U-Net with the possibility of deep learning in the convolutional neural network, is applied. The image segmentation results provide helpful information for the selection of a representative microstructure applied in FE simulations, the results of which will be presented in the consecutive work. Based on the results obtained, the following conclusions can be drawn:

- Samples that showed quasi-brittle behavior possess a global ultimate tensile strength of about 100–130 MPa higher than those with ductile behavior ($\sigma_{u}\approx$ 630 MPa).
- Measured local strains with high values locate around large WC particles but not around diamonds.
- The debondings between the diamonds and the (Co-WC) matrix, including their connecting, are the primary damage mechanism for the composite with the quasi-brittle behavior since no WC debonding is observed in most cases, even though some WC particles are even larger than diamonds. One of the investigated samples showed debonding between an extraordinary large WC particle (recalculated diameter about 620 μm, mean size 3–5 μm) and the matrix, i.e., it is taken as happening by chance and not the general case.
- Concerning the quasi-brittle damage evolution, some short cracks are formed due to void/debonding connections. Such crack formation locates among particular diamond clusters: (i) with short distances among diamond inside these clusters; (ii) with short distances to other clusters. Local morphology-dependent fatal fracture located at a region with a high diamond concentration (12 vol.% detected on the fracture surface compared to global 5 vol.%). This high concentration can be caused by the local feature uncertainty of commercial samples.
- Nanoindentation results reveal that the WC phase should possess higher phase stress (coincidence with high strain) than the diamond phase.
- From local strain maps, high strain values around the large WC particles are observed in the matrix for the composite with quasi-brittle behavior. However, no similar strain pattern is presented around diamonds. It is expected that the WC particles possess very high stresses, causing high matrix deformation around them. This possibly sheds light on that WC phase burdens more stress than diamonds.
- The U-Net method in ML takes only about 40 min to segment more than 700 images, i.e., a great improvement of the time efficiency compared to the manual work. From the results of autonomous data handling processes, we realized that the detected WC volume in the μCT images decreases with increasing scanning depth due to its fine size and the scattering of the X-ray energy.

One of the further works will be calculating strain maps using 3D DIC, which should provide more accurate data concerning the local deformation behavior.

## Figures and Tables

**Table 1:** The composition and particle mean sizes (in diameter) of the two types of Co/WC/diamond composites with 90 vol.% Co, which the manufacturer provided.

Type	Behavior	WC Phase		Diamond Phase	
		vol.%	Mean Size [μm]	vol.%	Mean Size [μm]
I [49]	ductile	5	3–5	5	90
II	quasi-brittle	5	3–5	5	40–50

**Table 2:** Material parameters for the diamond, the WC phase and Co matrix [49,50].

Phase	Young’s	Poisson’s	Thermal Expansion
	Modulus [GPa]	Ratio [-]	Coefficient [1/K]
Diamond	890	0.19	1.18 × 10^−6^
WC	707	0.20	5.20 × 10^−6^
Co	211	0.32	12.5 × 10^−6^

## Data Availability

Data is contained within the manuscript.
